# Permanent health education for health economics in Brazil: a forgotten facet?

**DOI:** 10.3389/fpubh.2026.1776536

**Published:** 2026-03-02

**Authors:** Manoel Honório Romão, Jordana Crislayne de Lima Paiva, Lorena de Macêdo Silva, Elinaldo Bernardo de Oliveira Junior, João Maria Macêdo da Costa, Israel José dos Santos Felipe, Rodrigo Pires de Campos, Thaísa Góis Farias de Moura Santos Lima, Maria Carmem F. D. Rêgo, Mônica Karina Santos Reis, Marilyn Anderson Alves Bonfim, Janaina Luana Rodrigues da Silva Valentim, Karilany Dantas Coutinho, Aline de Pinho Dias, Carlos Alberto Pereira de Oliveira, Karla Mônica Dantas Coutinho, Natalia Araújo do Nascimento Batista, Marcella A. Da Rocha, Erika Santos de Aragão, Jane Mary de Medeiros Guimarães, Susana Henriques, Claudia Miranda Veloso, Ricardo Alexsandro de Medeiros Valentim, António Manuel Quintas-Mendes

**Affiliations:** 1 Laboratory for Technological Innovation in Health (LAIS), Federal University of Rio Grande do Norte (UFRN), Natal, Rio Grande do Norte, Brazil; 2 Distance Education and eLearning Graduate Program, Open University of Portugal/University of Minho, Braga, Portugal; 3 Institute of International Relations (IREL), University of Brasilia (UnB), Brasilia, Brazil; 4 Development Society and International Cooperation Graduate Program, University of Brasilia (UnB), Brasilia, Brazil; 5 Ministry of Health, Brasilia, Brasilia, Brazil; 6 Education Graduate Program, Federal University of Rio Grande do Norte (UFRN), Natal, Rio Grande do Norte, Brazil; 7 Oswaldo Cruz Foundation, Manguinhos, Rio de Janeiro, Brazil; 8 Advanced Center for Technological Innovation, Federal Institute of Education, Science, and Technology of the State of Rio Grande do Norte, Natal, Rio Grande do Norte, Brazil; 9 Center for Global Studies at the Open University of Portugal, Lisbon, Portugal; 10 Institute of Human Education With Technologies, State University of Rio de Janeiro (UERJ), Rio de Janeiro, Brazil; 11 Sustainability and Development Graduate Program, Open University of Portugal, Lisbon, Portugal; 12 Institute of Collective Health, Federal University of Bahia, Salvador, Bahia, Brazil; 13 Federal University of Southern Bahia, Salvador, Bahia, Brazil; 14 Laboratory of Distance Education and eLearning, Universidade Aberta, Lisbon, Portugal; 15 GOVCOPP, CEG, ESTGA, Universidade de Aveiro, Aveiro, Portugal; 16 Open University of Portugal, Lisbon, Portugal

**Keywords:** Brazil’s national health system (SUS), health economics, health financing, health management, permanent health education

## Abstract

**Introduction:**

This article analyzes the presence and induction of Permanent Health Education (PHE) in Health Economics (HE) within the official documents of the Department of Health Economics and Development (DESID) of Brazil’s Ministry of Health (MoH).

**Methods:**

This is an exploratory and descriptive qualitative study, grounded in the document analysis of 126 records categorized according to the elements proposed by Williams and content analysis based on Bardin’s method (2016).

**Results:**

The findings revealed a lack of programmatic, budgetary, and strategic guidelines specifically aimed at PHE in Health Economics, as well as a restricted provision of specialized courses and limited integration with public health management policies. Comparative analysis with international experiences, including the United Kingdom, Belgium, and South Africa, demonstrates that the institutionalization of HE training fosters greater efficiency, equity, sustainability, and rationality in the use of public resources. The findings suggest that continuous technical qualification in this field constitutes an essential component for strengthening Brazil’s National Health System (SUS).

**Discussion:**

In this context, there is a clear need for the formulation of government policies that stimulate, incentivize, and expand PHE in Health Economics, leveraging the potential of technological platforms such as AVASUS and the Brazil Telehealth Program, both of which possess significant reach, adherence, and engagement across the national territory.

## 1 Introduction

Brazil’s National Health System (SUS), established by the Federal Constitution of 1988, has cemented its position as a universal public health model guided by the principles of universality, comprehensiveness, and equity (1). Despite the progress achieved throughout its history, persistent structural and operational challenges remain, notably concerning the efficient management of available resources amidst tightening fiscal constraints and escalating healthcare demands. In this scenario, Health Economics (HE) assumed a central role due to the constant growth of health expenditures and the pressure for more efficient, effective, and equitable systems. In Brazil, health spending accounts for about 9.6% of Gross Domestic Product (GDP), with public spending corresponding to nearly 3.8% (2).

Health economics is a strategic field of knowledge, as it combines economic principles with health practices. This field aims to promote greater allocative efficiency, financial sustainability, equity, and rationality in decision-making (3, 4). The literature demonstrates that HE has grown into an interdisciplinary field, supported by robust methodology, capable of integrating economic theory with health needs (37, 38). Such an integration is essential for Brazil’s SUS, which faces increasing financial and structural contingencies, aggravated by an aging population and rising rates of chronic diseases.

In Brazil, the Department of Health Economics and Development (DESID), under the Ministry of Health (MoH), plays a central role in this field. DESID’s mission is to provide economic analysis, develop tools to monitor budgetary execution and public procurement prices and costs, as well as to promote initiatives such as Health Economics Centers (5, 6). However, there is a critical gap in the application of these instruments; a plausible rationale is the lack of permanent health education (PHE) in the field of health economics. This shortcoming compromises the capacity to ensure more rational, sustainable, and evidence-based decisions.

The concept of Permanent Health Education (PHE), established by the National Policy on Permanent Health Education (PNEPS) through Ordinance No. 198/2004, serves as the theoretical foundation of this study (7). PHE is understood as a transformative strategy aimed at improving health services by fostering critical reflection, collective learning, and participatory practices within the health system (8, 9).

This approach transcends traditional, vertically oriented training models by emphasizing the integration of teaching, management, care, and social control (known as the “training quadrilateral”) as essential dimensions for developing professional competencies and promoting systemic change. Grounded in this conceptual perspective, the study adopts Williams’ analytical model (1987), which organizes health economics into eight elements, ranging from supply and demand to planning and systemic evaluation mechanisms.

International experiences in the United Kingdom, Belgium, and South Africa have shown significant progress in incorporating HE into educational policies from undergraduate level onwards and into permanent health education programs (10–12). In Brazil, however, training initiatives in this field are sporadic, fragmented, and restricted to a few institutions, thereby hindering their consolidation as a structural pillar of public policies within Brazil’s SUS (13). Given this scenario, this study sought to answer a key research question: to what extent has health economics been incorporated, or neglected, in PHE policies within Brazil’s SUS?

To answer this question, this study used official DESID documents and adopted the concept of Permanent Health Education (PHE) as its theoretical foundation, understood as a strategy for transforming work practices and improving health services by fostering critical reflection and collective learning within the health system (9, 14, 15). This approach recognizes education as a dialogical and continuous process that integrates professional development with the organization of work in healthcare settings, thereby aligning with the transformative principles of Brazil’s Unified Health System (SUS).

In this conceptual context, the study also adopted Williams’ analytical model (1987), which organizes health economics into eight elements, ranging from supply and demand to planning and systemic evaluation mechanisms. This model provided a structured analytical framework that guided the examination of official DESID documents and allowed for a systematic interpretation of the relationship between PHE and the institutional development of Health Economics within Brazil’s SUS.

Understanding the role of permanent health education in consolidating HE as a strategic field is therefore essential for expanding the technical capacity of Brazil’s SUS and overcoming contemporary challenges in public health management. Although the shortage of consistent training in health economics is hindering communication between managers, professionals, and researchers, the key point is to understand why this field is crucial for the future of Brazil’s SUS. While this study does not establish a direct causal link, the literature suggests that a shortage of qualified personnel in health economics may contribute to fragmented system operations. Theoretically, this gap poses challenges in coordinating resources and evaluating policy impacts, although further empirical research is needed to quantify these effects within the SUS.

In this sense, PHE for public health workers transcends mere technical improvement. It constitutes a strategic condition for ensuring the financial sustainability, organizational efficiency, and systemic effectiveness of the SUS. Developing skills in the field of HE allows the health system to respond in a more integrated and equitable way to the growing challenges facing Brazilian public health.

Finally, it is observed that the international scientific literature employs multiple terminologies to address the theme of Permanent Health Education. This polysemic diversity, which alternates between Permanent Education, Continuing Education, and In-Service Education, reflects distinct theoretical, political, and institutional frameworks. In light of this scenario, the adoption of the term Permanent Health Education is established as a political and conceptual choice, aligned with Brazil’s National Policy on Permanent Health Education, and is consistently used throughout this study.

## 2 Methodology

This article adopts a critical narrative review approach, combining document analysis and the authors’ experience as teachers and researchers in the field. The research consulted national and international bibliographic databases, as well as institutional documents from the Ministry of Health (MoH), the Pan American Health Organization (PAHO), and the Coordination for the Brazilian Federal Agency for Support and Evaluation of Graduate Education (CAPES). The objective was not to conduct an exhaustive survey, but rather to question the way in which the health economy has been structured in relation to the Continuous Health Education Policy in Brazil, comparing it with international experiences and suggesting future paths.

The research is characterized as exploratory and descriptive, with a qualitative approach. The methodological approach was developed in four stages: (1) document analysis of available public records; (2) content analysis with identification of thematic axes; (3) complementary mapping of courses and training programs; and (4) correlation of findings with the Health Economics framework proposed by Williams (16).

The research corpus consisted of 126 documents, including technical, regulatory, and institutional publications produced or endorsed by the Department of Health Economics (DESID/SECTICS/MS). These materials were indexed in the Virtual Library of Health Economics and the National Health Economics Information Portal. A comprehensive list of these documents is now provided in Table 1, which categorizes them by type, source institution, and year of publication to ensure full transparency and reproducibility of the analyzed corpus.

**Table 1:** Distribution of documents analyzed by gender, type, and quantity.

Document genre	Document type	Quantity
Judgment	Decision by the supervisory body	07
Legislation	Federal Constitution	01
	Decree	02
	Amendment	01
	Laws	04
	Ordinance	07
	Resolution	04
Spreadsheet	Spreadsheet	01
System	System	01
Bibliographic production	Article	14
	Bulletin	12
	Guideliness	04
	Scientific production catalog	01
	Thematic glossary	07
	Technical Guide	03
	Book	22
	Manual	25
	Report	04
Other publications	Document	02
	Home page	04

Source: Prepared by the authors.

To promote open science and facilitate future research, the complete dataset, containing the classified list of all 126 reviewed documents has been made publicly available in the Zenodo repository (DOI: 10.5281/zenodo.18472912).

The selection of documents followed strict eligibility criteria. Inclusion criteria were: (1) official documents (technical, regulatory, or institutional) produced or endorsed by DESID/MS; (2) materials available in the Virtual Health Library for Health Economics and the National Health Economics Information Portal; and (3) publications dated up to March 2024. Exclusion criteria were: (1) duplicate materials; (2) documents not directly addressing the economic management of the SUS or professional training in health; and (3) unsystematized content or materials without institutional authorship (e.g., opinion pieces). This systematic approach clearly delineates the scope and ensures transparency and reproducibility of the document analysis.

The searches were conducted between November 2024 and February 2025, and aimed to identify evidence of the induction of policies to promote permanent education through legal and administrative mechanisms in the field of Health Economics.

In the first stage of the research, a document analysis was conducted, understood as a systematic method for locating, reviewing, and interpreting printed and electronic documents (17, 18). The document collection was organized in a spreadsheet, categorized according to genre, type, and content.

Official materials linked to the Department of Health Economics (DESID) of the Brazil’s MoH were included, available until March 2024 in databases such as the Virtual Health Library and the National Health Economics Information Portal. The documents were classified into the following categories: judgments, legislation, spreadsheets, systems, and bibliographic production (including books, manuals, bulletins, articles, reports, primers, glossaries, scientific catalogs, and technical guides). Table 1 shows the distribution of documents according to document type, category, and quantity identified:

Data analysis was conducted using content analysis, defined as “a set of communication analysis techniques that aims to obtain, through objective and systematic procedures for describing message content, indicators (quantitative or otherwise) that allow inferences to be made about the conditions under which these messages are produced/received” (19), p. 33.

The content analysis followed the systematic stages proposed by Bardin (19). It began with a pre-analysis phase involving the organization of the corpus and the formulation of initial hypotheses. Subsequently, we conducted an in-depth exploration of the material, coding 126 documents according to the eight analytical elements of Health Economics defined by Williams (16). These elements (A–H) were applied as *a priori* categories to enable a structured classification of content. The final stage comprised the treatment of results, during which we carried out inference and interpretation to identify thematic patterns and semantic frequencies, thereby ensuring a rigorous and reproducible analytical process.

Based on this approach, the n-grams present in the textual corpus were extracted and analyzed using the TF-IDF (Term Frequency–Inverse Document Frequency) algorithm, as described by Da Rocha et al. (20). This method, recognized as a classic text mining technique, allows the relevance of terms or expressions to be quantified based on their local frequency and global rarity within the corpus. Thus, the algorithm was applied to systematically and objectively identify the most important n-grams in the analyzed data set. The results generated indicators that reveal semantic patterns and were subsequently represented using weighted word clouds and bar charts, promoting a clearer interpretation of the predominant content and broadening the understanding of the communicative dynamics present in the material studied.

For the interpretation of the findings, Williams (16) model was adopted as a theoretical reference, which proposes eight analytical elements of Health Economics. These elements provide a comprehensive framework for identifying how economic variables interact in the organization and functioning of health systems. They relate aspects such as supply, demand, health value, political decisions, planning, and evaluation of results. Eight elements were analyzed:

- A - what influences health? (other than health care);
- B - what is health? what is its value?;
- C - demand for health care;
- D - supply of health care;
- E - micro-economic evaluation at treatment level;
- F - market equilibrium;
- G - evaluation at whole system level e.
- H - planning, budgeting, & monitoring mechanisms (16).

Element (A) refers to factors that influence health beyond medical care, such as socioeconomic conditions, occupational risks, consumption patterns, education, and income. Element (B) deals with the definition and valuation of health, involving perceived attributes, utility scales, and quality of life indices. Element (C) addresses the demand for healthcare, influenced by elements (A) and (B), which is determined by barriers to access (price, time, psychological factors), the agency relationship between different actors (users, professionals, establishments, etc.), and the perception of need. Element (D) comprises the provision of health services, including production costs, delivery techniques, inputs, labor markets, and forms of remuneration.

Element (E) refers to microeconomic evaluation at the treatment level, through cost-effectiveness and cost–benefit analyses, covering decisions on the mode, location, timing, and intensity of care. Element (F) deals with market equilibrium and rationing mechanisms (monetary and non-monetary, such as waiting lists), which impact the distribution of services. Element (G) encompasses the health system as a whole, based on criteria of equity and allocative efficiency, allowing for intra- and international comparisons. Finally, element (H) covers planning, budgeting, and monitoring mechanisms, which are fundamental to system governance and the management of resources, standards, and incentives.

The research included official DESID documents from the Ministry of Health available in the Virtual Health Library (VHL) and the National Health Economics Information Portal, a collaborative portal managed by the Ministry of Health, which addresses topics related to the economic management of Brazil’s National Health System or professional training in health. Duplicate materials, materials irrelevant to the topic, or incomplete materials were excluded. The analysis focused on public and institutional documents, without including professionals’ perceptions, practical experiences, or unsystematized content, which constitutes an approach geared toward the interpretation of official sources.

In light of this guidance, a complementary mapping of public sources was conducted, including institutional platforms of universities and government schools, aiming to identify existing training initiatives and establish a correlation between the documentary findings and the actual availability of courses in the country. This procedure broadened the scope of the analysis, allowing for a comprehensive view of both the normative perspective (official documents) and the formative perspective (offered courses).

During the analysis process, a scarcity of records referring to the offering of courses or training programs directly linked to Health Economics was observed in the official documents analyzed. The results are presented below, structured according to Williams (16) eight analytical elements, to answer the following research questions, derived from the central question:

- RQ1—How does the Department of Health Economics and Development (DESID) of the Brazilian Ministry of Health (MoH) incorporate the need for education in health economics into the implementation of its actions, especially for policymakers and managers of the Brazilian National Health System (SUS)?
- RQ2—Does the Department of Health Economics and Development (DESID) of the Ministry of Health (MoH) consider the potential effects of permanent education in Health Economics on the development of the field and on public health management?

## 3 Results

From the documents that were analyzed, the main themes emerged: Socioeconomic factors; Definition and valuation of health; Demand for healthcare; Provision of health services; Microeconomic evaluation of health; Health market equilibrium; Equity and efficiency of the health system, and Planning, budgeting, and monitoring mechanisms, which structure the results obtained.

The first focus area addresses the financing and sustainability of the healthcare system, reflecting the importance of ensuring long-term financial viability. This axis also covers the need to optimize the use of available resources and reduce environmental impacts. The second axis focuses on management, transparency, and monitoring. Several documents show the importance of strengthening mechanisms that ensure accountability and quality in the administration of the Health System.

Tools such as the Public Health Budget Information System (SIOPS), Health Price Database (BPS), Federal Government Materials Catalog (CATMAT), and ApuraSUS have a fundamental role in standardizing health economics processes in the Brazilian National Health System. These tools aim to establish uniform, comparable, and transparent technical criteria to support management, regulation, and social control, transparent access to information, efficient use of resources, and cost management, which ensures continuity of services for the population. Economic and technological health assessments appear repeatedly in the documents, highlighting the importance of considering costs and effectiveness in incorporation decisions.

**Figure 1:**
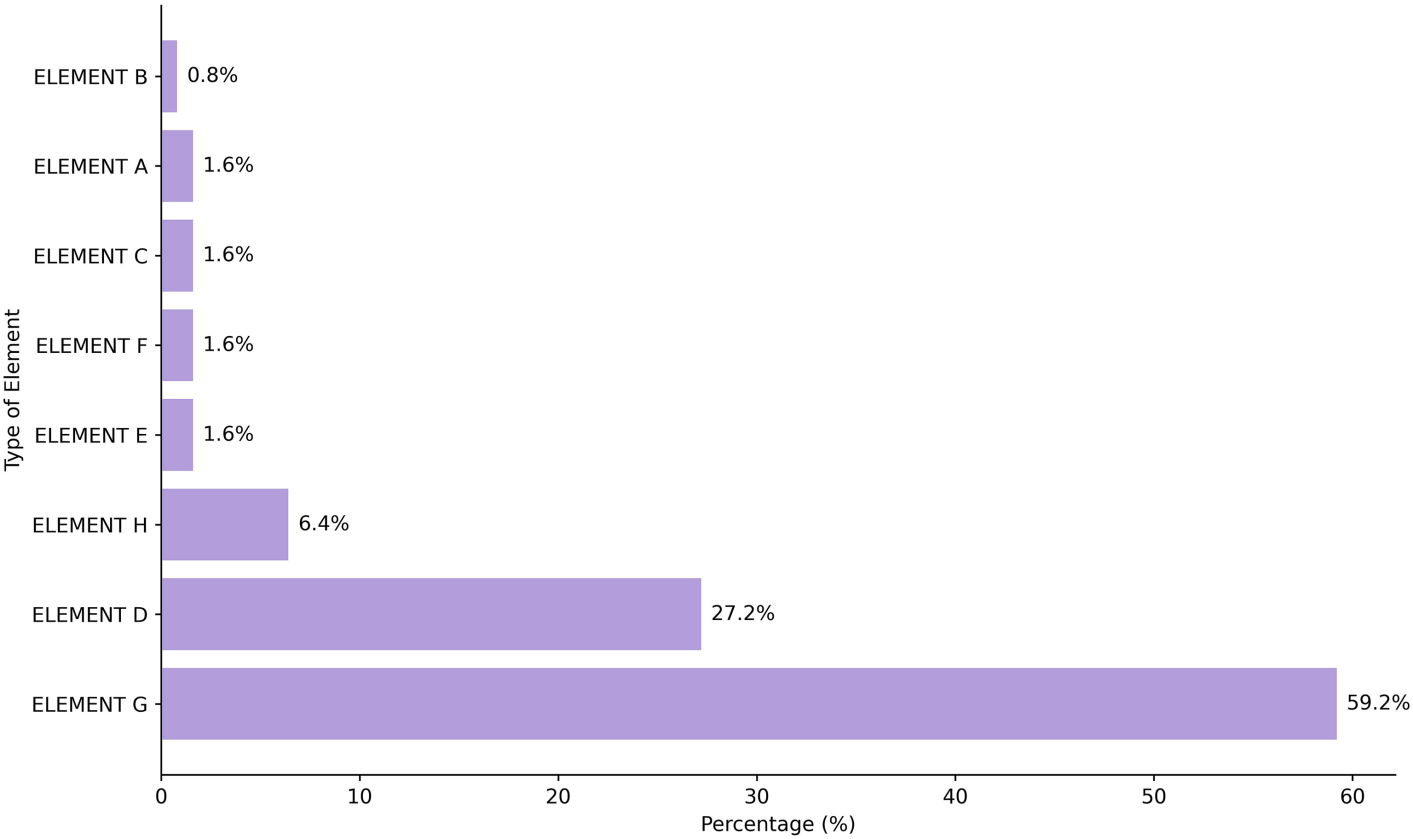
Percentage distribution of documents in Williams' (16) Elements of Health Economics. It is worth recalling what is explained in the methodology of this research, based on Williams’ model (1987), which proposes eight analytical elements of Health Economics: A, What influences health (other than health care)?; B, What is health? What is its value?; C, Demand for health care; D, Supply of health care; E, Micro-economic evaluation at treatment level; F, Market equilibrium; G, Evaluation at whole system level; and H, Planning, budgeting, & monitoring mechanisms.

Another thematic axis identified refers to training and dissemination of content for managers and professionals working in public health in Brazil, using health economics methodologies and tools, which show the need to improve training and expand access to information made available by Brazil’s MoH. The fifth axis highlights the public policy management model, which addresses the need for innovation and development in the administration of the Brazilian National Health System (SUS). Topics related to health service planning, post-pandemic health policies, and SUS management models were frequently discussed to organize the system more efficiently and equitably. Figure 1 shows the representation of these axes in the analyzed document corpus.

When comparing the thematic axes identified in this study with the elements proposed by Williams for economic analysis in health, a significant predominance of element G, “evaluation at whole system level,” is observed, present in 59.2% of the documents analyzed. This element assesses the overall performance of the health system, involving criteria of allocative efficiency, equity, quality, and sustainability, which enables comparative analyses between regions and countries. This emphasis is consistent with the themes identified in the research, especially those related to the financing and sustainability of the health system, management and transparency, and the public policy management model, which reflect concerns about the rational use of resources and the efficient organization of the Brazilian National Health System at the global level.

Although elements E (microeconomic evaluation) and F (market equilibrium mechanisms) appear less frequently in the documents analyzed, their inclusion in permanent education processes is essential due to the possibility of enabling more sustainable decision-making without compromising equity or quality of care. Additionally, it prepares professionals to face real-life dilemmas, particularly in the context of the Brazilian National Health System. The analysis indicates that the training content currently offered in Brazil prioritizes a systemic view (element G), but neglects technical training in cost-effectiveness analysis, cost–benefit analysis, resource rationing, and efficient resource allocation, all of which are essential components for the practical work of health managers and professionals.

Williams’ model does not propose a rigid hierarchy among the eight analytical elements of Health Economics, but understands that they are parts of an interconnected system, in which each element contributes to the understanding of different aspects of the health sector. Therefore, despite the absence of an explicit order of importance, a coherent structural logic permeates the different levels of analysis. As such, permanent education can be seen as an instrument, catalyst, and articulator capable of integrating the skills necessary for applying the three elements at different levels of public management. However, the current landscape of Permanent Education in Health Economics in Brazil reveals a departure from this integrative proposal.

The survey identified a limited and scattered offering of courses focused on this type of training. The initiatives found were classified into three main categories: free and introductory courses, university extension courses, and postgraduate programs (specialization, master’s, and doctorate). In total, 10 programs were mapped based on related themes, with significant variation in terms of modality, workload, scope, and target audience.

Although the survey identified 10 training initiatives with topics related to Health Economics, not all of them directly address the core technical and economic content of the field. Some of the courses focus more on public management, the organization of the Brazilian National Health System, and health financing, without delving into the analytical and methodological aspects specific to health economics, as defined in elements E, F, and G proposed by Williams (16). Only a portion of the initiatives, such as the courses offered by the Oswaldo Cruz Foundation (Fiocruz), the Federal University of Goiás (UFG), the Federal University of Bahia (UFBA), the Federal University of Pernambuco (UFPE), and the University of Brasília (UnB), present a more specific and structured approach to the topic. However, these offerings are insufficient in terms of reach, quantity, and training frequency. In this process, it is necessary to consider the territorial aspects of Brazil, which comprises 26 states, one Federal District, and more than 5,500 municipalities, as well as the vast contingent of health workers employed by the Brazilian National Health System (SUS), numbering over 4 million professionals. Studies show that technology-mediated health education initiatives have achieved this goal on a broad scale, strengthening professional qualifications nationwide (21, 22).

Among the free courses, we highlight “Political Economy of Health for All,” promoted by Fiocruz, with a workload of 45 h and accessible language, as well as the courses “Management of Public Policies at the Local Level” and “Public Health Policies,” offered by the National School of Public Administration (ENAP) and aimed primarily at municipal public officials. These initiatives, mostly in the form of distance learning (DL), are introductory in nature and open to the general public, which facilitates access, but with limited scope in terms of technical and scientific depth.

In the extension category, initiatives such as the 20-h course “Health Economics Topics” offered by the Open University of Brazil (UAB) at the Federal University of São Paulo (Unifesp) and the extension course “Health Economics” promoted by the Federal University of Bahia (UFBA) through the Institute of Collective Health (ISC) were identified. These courses seek to deepen specific knowledge in the field and play an important role in the technical qualification of active professionals. However, they are sporadic, have limited regional coverage and are not offered permanently.

At the postgraduate level, four specializations were found. In Brazil, lato sensu postgraduate courses are courses for people who need to specialize in a specific area, but they are not at the master’s or doctoral level, with a direct focus on Health Economics, offered by institutions such as the University of São Paulo (USP), University of Brasília (UnB), Federal University of Goiás (UFG), and Federal University of Pernambuco (UFPE). Some of these initiatives are the result of partnerships with Brazil’s MoH, such as the UFG course, conducted in conjunction with the Institute for Health Technology Assessment (HTA). In addition, the Federal University of Pernambuco offers a master’s and professional doctorate program focusing on Health Management and Economics, and elective graduate courses offered by the Federal University of Minas Gerais (UFMG).

Despite the relevance of these programs, it is observed that the offer is concentrated in a few institutions, often with access restrictions, and limited coordination with national permanent education policies. The perceived low coordination between training programs and DESID is based on the absence of formal integration strategies or joint guidelines within the analyzed documents. The lack of documented partnerships in strategic plans and management reports suggests a normative gap in the institutionalization of these educational initiatives.

The lack of systematic, integrated programs with national coverage reinforces the need for strategic planning to consolidate Permanent Education in Health Economics as a structural axis for the qualification of professionals in the Brazilian National Health System (SUS). This reinforces the finding that training in Health Economics in Brazil is still sporadic, fragmented, and disconnected from public health policies. It is also predominantly introductory, which compromises its role as a structuring axis and limits its potential as a strategic component of permanent education for SUS managers and professionals. This last aspect indicates the fragile inductive capacity of the programs, projects, and actions promoted by DESID.

Even with specific initiatives mapped out, analysis of the documents reveals the absence of a structured, systematic, programmatic, continuous policy with resource provisions focused on permanent education in Health Economics. The lack of clear funding guidelines for continuing education programs compromises the institutionalization of this area as a strategic axis for strengthening and ensuring the sustainability of the Brazilian National Health System (SUS).

The gap in budget planning hinders the expansion of educational offerings and limits the national reach of existing initiatives, contributing to the fragmentation and discontinuity of educational actions that challenge work processes and link teaching and intervention in the reality of the territories. This phenomenon helps explain some scenarios of resource waste in the SUS, particularly in the National Immunization Program (PNI), which has repeatedly stood out for recording billions in losses.

**Figure 2:**
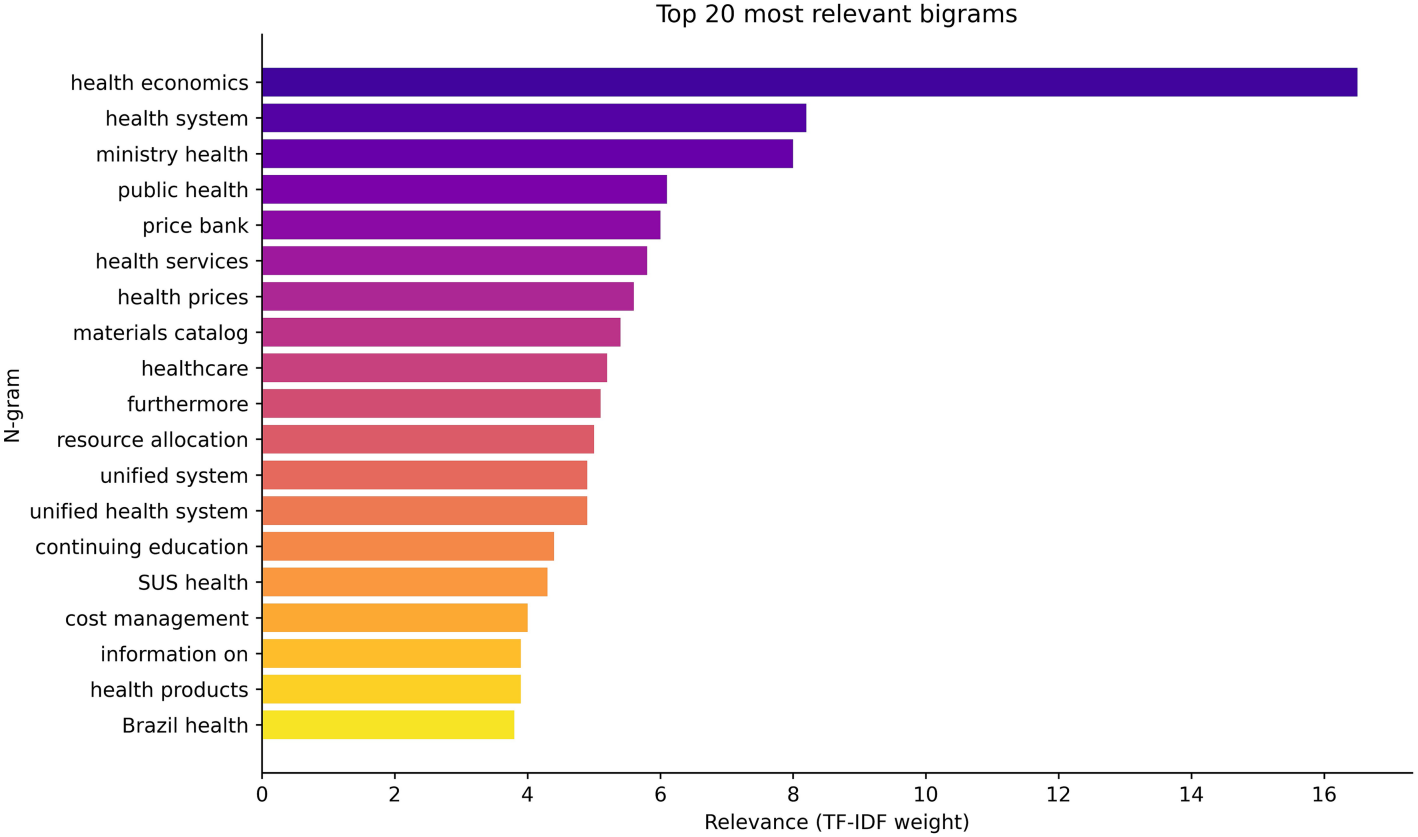
Bigrams of relevance in health economics and permanent education in health.

**Figure 3:**
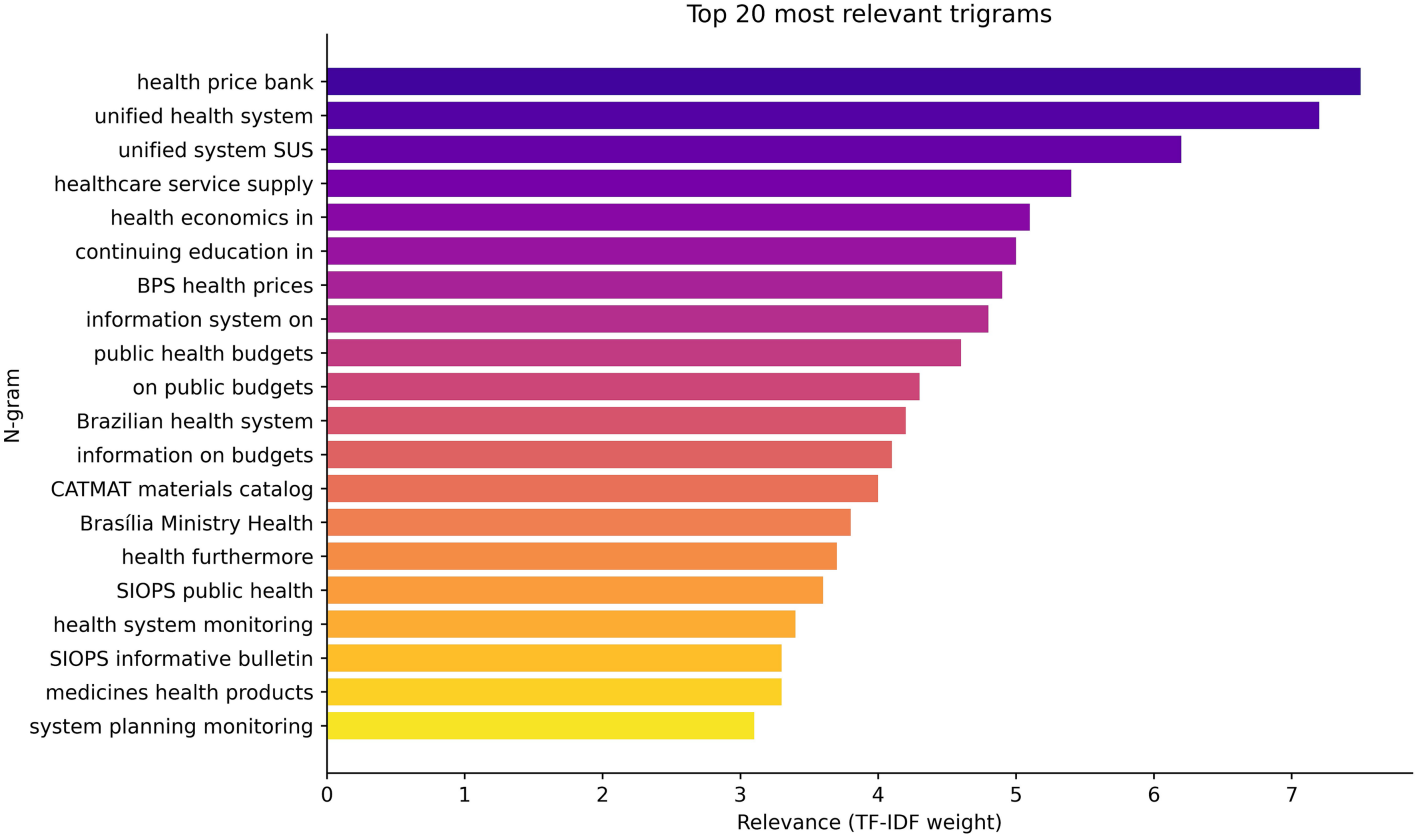
Relevant trigrams in health economics and permanent education in health.

The n-gram analysis showed, as shown in Figures 2, 3, that despite the conceptual relevance expressed in terms such as “health economics,” “permanent education,” “permanent education in,” “cost management,” and “resource allocation” having a strong presence and semantic articulation in the corpus studied, there is no materiality of these categories translated into policies, guidelines, or systematized practices within the Department of Health Economics, Investments, and Development (DESID).

The results of the exploratory and descriptive research demonstrated, first, the absence of structured guidelines to guide permanent education in Health Economics. The document analysis revealed that there is no programmatic, budgetary, or strategic policy focused on permanent education, nor are there any financing mechanisms or resource forecasts to support ongoing educational initiatives. This gap compromises the institutionalization of permanent education as a structuring axis of DESID’s activities. In other words, the incorporation of the need for permanent education in Health Economics into the actions institutionalized by the Department remains fragile, disjointed, and insufficient.

In addition, the programs and projects conducted by DESID were found to have low capacity to induce change in terms of training managers and professionals. The fragility of training activities compromises coordination between managers, workers, and researchers, reducing the pedagogical and strategic power of the economic analyses produced by the area.

Another key finding concerns the limited, sporadic, and scattered nature of training opportunities in health economics. Existing initiatives are sporadic, with low regional coverage and limited technical and scientific scope, and are unable to meet the training needs of a system with more than 4 million workers distributed nationwide. This limitation directly affects the ability of SUS managers to use analytical and economic tools critically and in an applied manner.

Finally, there was a significant gap in the practical application of tools developed by DESID, such as the Public Health Budget Information System (SIOPS) and the Health Price Database (BPS). The findings indicate a potential gap in their optimal use, primarily due to the absence of continuous training protocols in the official documents. This suggests that, while these systems are prominent, the institutional framework for qualifying managers to operationalize them remains insufficient.

Thus, the findings indicate that, although the relevance of Permanent Education in Health Economics is recognized discursively and evidenced by the linguistic patterns of n-grams, this importance is not converted into consolidated policies, investments, or institutional strategies on the part of DESID. This disconnect weakens the MoH’s ability to strengthen the economic governance of the Brazilian National Health System and to incorporate evidence-based practices into the decision-making process.

## 4 Discussion

The document analysis revealed weaknesses in the promotion of PHE in the field of health economics, marked by the absence of coordinated strategies for continuing technical training within Brazil’s SUS. Although HE is widely recognized as crucial in the efficient management of SUS, the documents analyzed do not present a coordinated strategy that aligns permanent technical training, intertwined with the PNEPS, with SUS planning and management guidelines.

Williams’ theoretical model (1987) structures HE into eight interconnected elements that, when applied to public policy analysis, provide a comprehensive framework for evaluating different aspects of health systems. This model made it possible to structure the document analysis, so as to understand to what degree the elements of HE feature and interact in the training and management strategies discussed in the materials examined.

In addition, the documents were interpreted in light of the theoretical assumptions of permanent health education, which aims to transform professional practices and continuously improve services. Hence, the contents were systematized, classified, and analyzed in order to identify the degree of training instilled in institutional initiatives focused on SUS management.

Currently, PHE has been considered an innovative strategy in the context of Brazil’s SUS, capable of overcoming the limitations of traditional continuing education. PHE can transform professional practices, improve the quality of services, and strengthen daily work as an educational and collective space that permeates concrete relationships. Furthermore, PHE fosters dialog, reflection, and evaluation among workers, managers, professors, as well as all the users of healthcare initiatives and services (23). Nevertheless, in the particular case of Health Economics, significant gaps have been identified both in the provision and integration of training with current public policies. The absence of a structured approach to this field compromises managers’ ability to use economic evaluation tools for the efficient allocation of resources.

The consequences of the scarcity of professional education are evident: decisions regarding financing, procurement, technology assessment, and financial sustainability tend to lack a solid technical foundation, directly impacting the system’s efficiency and equity. This vulnerability is heightened by barriers to accessing educational programs, as most identified courses are one-off initiatives with restricted regional scope, limited hours, and narrow target audiences.

The analysis also highlighted the absence of clear budgetary guidelines for PHE in Health Economics. This budgetary gap reflects the low political priority given to the field and compromises the implementation of more robust strategies with national reach. The lack of structured investment prevents the expansion of training programs and limits the impact of PHE on public management.

On the other hand, international studies indicate that countries investing in the institutionalization of Permanent Health Education (PHE) in Health Economics have achieved substantial gains in efficiency and sustainability. The assertion that investing in Health Economics (HE) training leads to efficiency gains is supported by studies such as those by Folland et al. (24), who emphasize that economic literacy among health professionals reduces resource waste and improves resource allocation. Furthermore, Jain (10) demonstrates that integrating HE into professional curricula enhances technical capacity for evidence-based decision-making, directly impacting system sustainability.

In this regard, the United Kingdom (UK) stands out as a global benchmark by systematically integrating HE principles into the clinical, educational, and policy decisions of its public health system. The National Institute for Health and Care Excellence (NICE) is an emblematic example. In the evidence and incorporation process, it utilizes, among other tools, economic evaluations to guide decisions on the incorporation of technologies and therapies into the National Health Service (NHS), promoting the efficient use of resources based on cost-effectiveness criteria and population impact (25).

While the NICE model remains internationally recognized as a methodological benchmark for cost-effectiveness and technology assessment, recent analyses highlight that the NHS has faced persistent challenges related to access, funding, and quality of care. This demonstrates that economic evaluation tools are valuable for prioritization but cannot alone ensure system performance without adequate structural and financial support.

In the UK, the education of healthcare professionals in economic knowledge is encouraged from the undergraduate level, as highlighted by the literature on teaching the discipline in British medical schools (10, 26). This approach strengthens decision-making based on clinical and economic evidence.

Furthermore, a study by Gray & Lorgelly (27) indicated that students taught by health economists achieved superior performance compared to those instructed by professionals from other fields, suggesting that the involvement of specialists is a determining factor for the quality of education in this area. The authors also concluded that the learning environment and students’ knowledge levels vary significantly across institutions, reinforcing that the provision of teaching by specialized economists can be an effective strategy to enhance professional education in Health Economics (27).

A report from the University of York (28) presents three essential concepts of economic evaluation—opportunity, perspective, and the cost–benefit margin—which have been widely disseminated within the British health system for promoting more rational and transparent resource allocation decisions. This model has served as a benchmark for other countries seeking to integrate health economics into both professional education and public management.

However, it is important to acknowledge that this model faces significant contemporary challenges, with recent literature highlighting tensions between economic efficiency and maintaining universal access and quality of care within the NHS.

In Belgium, the Belgian Health Care Knowledge Center (KCE) conducts systematic economic evaluations and promotes training programs focused on technology incorporation and cost-effectiveness analysis, directly influencing public policy planning (12). The success of these initiatives stems from the coordination between government institutions, universities, and research centers.

In South Africa, continuing professional development policies have promoted the strengthening of healthcare professionals’ competencies, especially in vulnerable regions. PHE programs based on educational technologies have been used as tools for workforce qualification and retention within the health system. Studies indicate that these strategies have a positive impact on management efficiency and healthcare delivery, particularly in the fields of HIV and tuberculosis (11).

These international experiences demonstrate that the institutionalization of PHE in Health Economics not only raises the technical quality of managers but also enhances the performance of public systems through improved resource allocation. In Brazil, the absence of educational and budgetary guidelines highlights a gap in this field and reinforces the need to establish a structured national strategy for PHE in Health Economics as a key component for the qualification of SUS management and the sustainability of public policies.

International literature underscores that PHE in health economics provides essential tools for cost assessment, trade-off analysis, and resource prioritization (24, 29). Recent evidence indicates that health education and literacy programs contribute to increasing the confidence, professional applicability, and communication skills of health workers, fundamental aspects for negotiation processes, technology incorporation, and accountability to society.

Røe et al. (30) show that pedagogical approaches to health literacy in higher education foster the acquisition of critical and communication competencies; Røe et al. (30) show that pedagogical approaches to health literacy in higher education foster the acquisition of critical and communication competencies; and Mendonça and Sousa (31) highlight that digital and media literacy is decisive in preventing misinformation and strengthening the resilience of health systems. These findings reinforce the relevance of structured PHE policies in the field of health economics as well, with impacts that extend beyond technical knowledge to improvements in health management and governance.

The ongoing qualification of professionals in health economics contributes directly to improved decision-making in public management by providing technical instruments that allow for the assessment of costs, impacts, and benefits of health policies, technologies, and interventions. This fosters more rational, transparent, and evidence-based decisions, mitigating resource waste and bolstering the effectiveness of implemented policies.

By promoting expenditure rationalization, optimizing resource allocation, and strengthening a culture of planning, PHE in health economics emerges as a pivotal strategy for the long-term sustainability of the public health system, particularly within contexts of fiscal constraint and escalating demand for services. The absence of such professional education represents not merely an educational gap but a barrier to efficiency, equity, and the consolidation of SUS principles.

Addressing the research questions posed, it is observed that DESID incorporates the need for PHE in health economics in a predominantly conceptual manner, lacking a corresponding materialization into structured institutional initiatives. Regarding RQ1, although official documents demonstrate the centrality of terms related to professional education—as evidenced by the high recurrence of expressions associated with health economics, PHE, and elements linked to cost management and resource allocation—, the document analysis revealed that this relevance does not translate into programmatic, budgetary, or strategic guidelines capable of sustaining a continuous qualification policy.

The absence of coordinated initiatives, coupled with the low inductive capacity of DESID’s interventions, results in fragmented and isolated educational offerings with limited reach; this, in turn, compromises the ability of managers and policymakers to effectively utilize essential instruments developed by DESID itself, such as SIOPS and the Health Prices Database (BPS). Consequently, the insufficiency of systematic PHE processes undermines the proficient application of these tools and weakens day-to-day management within the SUS context.

Regarding RQ2, the findings indicate that DESID recognizes, albeit implicitly, the potential effects of PHE on the development of the HE field and the advancement of public management. The analyzed documents suggest that PHE is understood as a fundamental element for bolstering financial sustainability, optimizing allocative efficiency, and ensuring the proficient and informed nature of decision-making.

The recurrence of terms associated with governance, planning, budgeting, and monitoring suggests that DESID perceives PHE as a constitutive element of the analytical and operational capacity required by SUS managers. However, the absence of institutional mechanisms to formalize and structure this process constrains the concrete realization of these effects, underscoring the imperative to institutionalize a consistent and nationally coordinated professional education program in health economics.

## 5 Conclusion

This study analyzed the official documents of the Department of Health Economics and Development (DESID) of Brazil’s MoH, aiming to identify the presence and induction of PHE in Health Economics. The analysis revealed a lack of a coordinated and continuous educational strategy, which hampers the consolidation of this field as a strategic component for the sustainability of Brazil’s SUS. Among the key findings, the following stand out: (i) the absence of clear budgetary guidelines for PHE; (ii) the limited and fragmented provision of courses, characterized by a restricted technical-scientific scope; (iii) poor coordination between educational and health management policies; and (iv) the concentration of initiatives within specific institutions, lacking national scalability.

From a practical standpoint, the results indicate that technical qualification in Health Economics can foster more rational, equitable, and efficient decision-making, reinforcing the fair allocation of public resources. In this context, PHE emerges as an indispensable tool for improving management in times of fiscal constraint and the growing demand for healthcare services. However, the research also presents limitations that may be mitigated in future studies. As a document analysis, it is limited to the content available through official channels and by the absence of explicit guidelines in the evaluated texts. Furthermore, the study did not consider the perceptions of professionals who are trained and operating in the field.

As a future agenda, it is recommended to evaluate existing training programs, map the needs of managers and professionals within Brazil’s National Health System (SUS), and expand comparative analysis with international experiences. A strategic measure would be the creation of a national observatory for Health Economics training, capable of monitoring course provision, identifying regional gaps, and aligning professional education with the system’s priorities. Coordination with established platforms—such as AVASUS (32), UNA-SUS, VCPH/PAHO, and OpenWHO—could broaden the reach and ensure greater integration between universities, government schools, and managers.

Based on the findings, there is a reinforced need to integrate PHE in Health Economics into public policy planning, recognizing its strategic role in the development of Brazil’s SUS. The absence of structured incentives and the low institutional priority given to this training reveal a misalignment between the complexity of SUS management and the available technical qualification.

Among the identified challenges, funding barriers, the scarcity of integrated programs, and a lack of specialized academic infrastructure stand out. Conversely, there are concrete opportunities for innovation and the expansion of such training, particularly through platforms and virtual learning environments (VLEs) within public universities, which can broaden the reach of technical qualification on a national scale by utilizing active methodologies and educational technologies.

From a global perspective, PHE in this field contributes to aligning Brazil’s SUS with the 2030 Agenda, particularly reinforcing Sustainable Development Goals (SDGs) 3 (Good Health and Well-being), 4 (Quality Education), 10 (Reduced Inequalities), and 16 (Peace, Justice, and Strong Institutions). This Permanent Health Education enables the integration of economic rationality, social equity, and efficiency in public management—essential aspects for achieving the SDGs. As noted by Ban Ki-moon, then UN Secretary-General, “The 2030 Agenda is a plan for people, planet, and prosperity. A universal roadmap that leaves no one behind.” (33).

Within the Brazilian context, the Ministry of Foreign Affairs itself recognizes that the 2030 Agenda “corresponds to a set of programs, actions, and guidelines that steer the work of the United Nations and its member states toward sustainable development”. PHE within Brazil’s SUS, as recommended by the Pan American Health Organization (2024), is linked to the promotion of health, equity, and social justice. Concurrently, by promoting the development of relevant skills, educational inclusion, and lifelong learning, it directly addresses the targets of SDG 4—specifically 4.3, 4.4, and 4.7—as defined by the 2030 Agenda.

Thus, it is imperative that Brazil moves toward the creation of a national training program or intervention in health economics, coordinating universities, teaching hospitals, state health departments, and Brazil’s MoH. This initiative should mandate the inclusion of health economics content within undergraduate programs in medicine, nursing, pharmacy, public administration, and hospital management, as well as PHE programs aimed at Brazil’s SUS managers, grounded in massive-scale and lifelong learning (21).

With qualified professionals, it will be possible to ensure decision-making that is more rational, transparent, and aligned with the principles of universality, comprehensiveness, and equity that guide Brazil’s SUS. Future investigations could map current HE competencies among managers and clinicians, so as to identify specific gaps and guide more effective training programs.

The Brazilian health reform movement has historically affirmed that health is a public good and a right of all, not a commodity. This principle also appears in the thinking of intellectuals such as Maria da Conceição Tavares, who emphasized that macroeconomic indicators do not directly translate into social reality. As she succinctly put it, “no one eats GDP, people eat food” (34).

Drawing inspiration from this reasoning, it is essential to recognize that, in the field of Health Economics, no one is healthy because of GDP, but rather as a result of efficient and equitable investments in health. This perspective echoes the principles reaffirmed by Brazil’s 16th National Health Conference, which emphasized that social well-being and health equity depend on democratic governance and fair resource allocation (35).

In a complementary perspective, Amartya Sen reminds us that economic development cannot be measured solely by statistical growth, but must be assessed in terms of human freedoms and capabilities. He cautioned that “we must not look only at statistical connections, but also evaluate and scrutinize minutely the causal processes that are involved in economic growth and development” (36), p. 15.

This reflection reinforces that the ultimate goal of Health Economics is not the maximization of efficiency indicators, but the expansion of people’s real opportunities to live healthy, dignified, and autonomous lives. In this sense, strengthening permanent health education in Brazil’s SUS represents a strategic path toward ensuring that economic rationality serves the ethical purpose of human development and social justice.

The contribution of this study to the field of Health Economics is twofold. First, it highlights the persistent training gap that exists in a country with Brazil’s characteristics. Second, it positions the coordination between Permanent Health Education (PHE) and health management as a structural pillar for the sustainability of Brazil’s SUS, the largest public health system in the world. Strengthening PHE in Health Economics is essential for consolidating more efficient, transparent, and evidence-based policies, thereby improving the allocation of public resources and ensuring the long-term sustainability of the health system.

## Data Availability

The original contributions presented in the study are included in the article/supplementary material, further inquiries can be directed to the corresponding author.
